# Association between thyroid function and diabetes peripheral neuropathy in euthyroid type 2 diabetes mellitus patients

**DOI:** 10.1038/s41598-023-40752-y

**Published:** 2023-08-18

**Authors:** Qingyuan He, Zekun Zeng, Man Zhao, Banjun Ruan, Pu Chen

**Affiliations:** 1 Department of Endocrinology, The First Affiliated Hospital of Xi’an Jiaotong University, Xi’an, 710061 Shaanxi People’s Republic of China; 2 Clinical Laboratory, Xi’an NO. 1 Hospital, Xi’an, 710002 Shaanxi People’s Republic of China

**Keywords:** Endocrine system and metabolic diseases, Diabetes, Peripheral neuropathies

## Abstract

Previous studies disclosed that a high thyroid stimulating hormone level is an independent risk factor for diabetes peripheral neuropathy (DPN) in subclinical hypothyroidism (SCH) patients with type 2 diabetes mellitus (T2DM). However, whether thyroid metabolism has an effect on DPN in euthyroid T2DM patients remains unknown. The aim of this study was to identify the association between thyroid function and DPN in euthyroid T2DM patients. A set of 580 euthyroid T2DM patients was enrolled in the current study and stratified into DPN and Non-DPN groups. Mann–Whitney U test was performed to analyze the continuous variables of biochemical and thyroid metabolism indicators, and the Chi-square test was used to compare the categorical variables. Spearman correlation analysis was performed to analyze the relationship between clinical indicators and free thyroxine (FT4). By using the logistic regression analysis, the prevalence of DPN in different thyroid function indicators were evaluated. T2DM patients with DPN had obviously lower levels of aspartate aminotransferase (AST), alpha-hydroxybutyric dehydrogenase (α-HBDH), superoxide dismutase (SOD), calcium (Ca), creatinine (Cr), uric acid (UA), retinol binding protein (RBP), total protein (TP), albumin (ALB), alanine aminotransferase (ALT) and FT4 than the T2DM patients without DPN (*P* < 0.05). FT4 was associated with TP, prealbumin (PA), ALB, SOD, anion gap (AG), Ca, chlorine (Cl), UA, RBP, apoprotein A (Apo A), apoprotein B (Apo B), apoprotein E (Apo E), and total cholesterol (TC). According to the FT4 quartile, participants were sequentially divided into four groups to compare the prevalence of DPN between each group. The data suggested that the prevalence of DPN in these four groups was 53.79%, 53.28%, 54.97%, 38.10%, respectively. Moreover, compared with quartile 4, patients in quartile 1, 2, 3 all had a significantly higher risk of DPN (*P* = 0.007, *P* = 0.011, *P* = 0.004). The level of FT4 was negatively correlated with the prevalence of DPN in euthyroid T2DM patients.

## Introduction

Diabetes is a lifelong metabolic diseases caused by multiple factors and characterized by chronic hyperglycemia, which is also an increasingly serious social and epidemiological problem. Among the diabetes patients, type 2 diabetes mellitus (T2DM) patients account for more than 90%. Hyperglycemia and metabolic disorders lead to acute and chronic diabetic complications, especially chronic complications are the main cause of diabetes disability and death. In patients with T2DM, up to 10–20% of them are suffering from diabetes peripheral neuropathy (DPN) at the time of diagnosis^1^. The main clinical manifestations are symmetrical sensory and motor disorders in the distal extremities. With the progress of diabetes, some patients may suffer from needle-like pain or other abnormal forms of pain in nerve endings, which develops into painful DPN, seriously affecting the life quality of T2DM patients. DPN has been identified to be associated with diabetic foot ulcers, infections, gangrene, and even amputation. Therefore, DPN acts as a risk factor for death and disability in diabetics. Oxidative stress, mitochondrial dysfunction and lack of nerve growth factor caused by metabolic disorders are implicated with the pathogenesis of DPN. However, the exact pathogenesis of DPN is still unclear, leading to a lack of effective treatment strategies in clinic.

Previous studies identified the risk factors of DPN including age, smoking, body mass index (BMI), the duration of diabetes, diastolic blood pressure (DBP), and other indicators e.g. glycosylated hemoglobin A1c (HbA1c), fasting plasma glucose (FPG) and, blood urea nitrogen (BUN) biochemical indexes^2–8^. In addition, a recent study reported that serum free triiodothyronine (FT3) level was correlated with nerve conduction in diabetes^9^, and other studies concluded that T2DM had an increased risk of DPN when TSH concentrations were higher^10^. Therefore, the aim of this study is to investigate the risk factors of DPN in T2DM patients with normal thyroid function, and analyze the relationship between thyroid function and DPN in these patients.

## Materials and methods

### Patients

**Figure 1:**
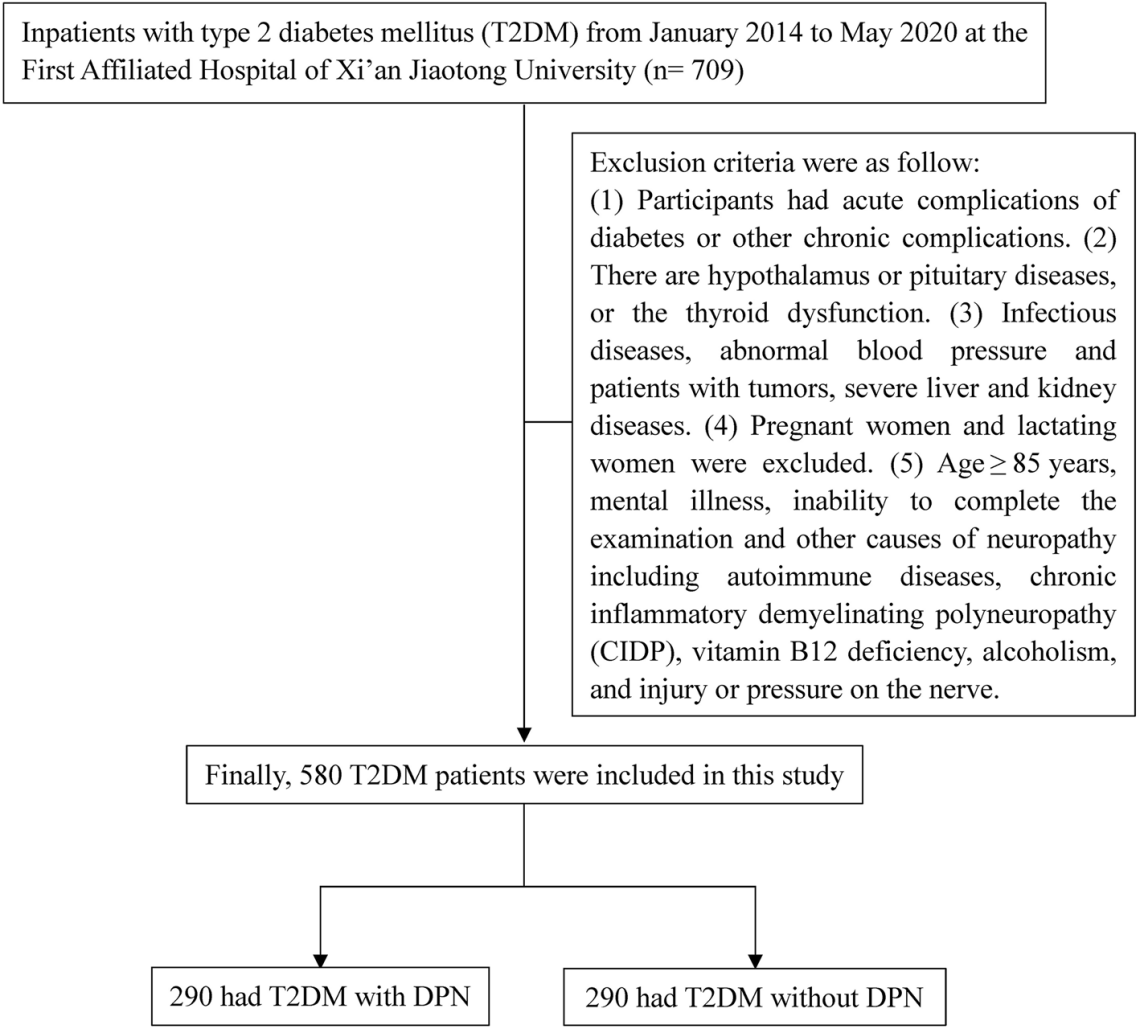
A total of 709 patients were recruited, and 129 patients were excluded. Finally, 580 T2DM patients were included in this study. Among them, there were 290 T2DM patients with DPN and 290 without DPN.

Inpatients with type 2 diabetes mellitus (T2DM) were enrolled from January 2014 to May 2020 at the First Affiliated Hospital of Xi’an Jiaotong University and valid data were collected from their medical records. During this period, a total of 709 patients were recruited. Diagnostic criteria for T2DM were based on the World Health Organization (WHO) diagnostic criteria for type 2 diabetes mellitus^11^, and the diagnostic criteria for DPN followed the 2010 European Association for the Study of Diabetes diagnosis expert consensus standards^12^. Exclusion criteria were as follow: (1) Participants had acute complications of diabetes or other chronic complications. (2) There are hypothalamus or pituitary diseases, or the thyroid dysfunction. (3) Infectious diseases, abnormal blood pressure and patients with tumors, severe liver and kidney diseases. (4) Pregnant women and lactating women were excluded. (5) Age ≥ 85 years, mental illness, inability to complete the examination and other causes of neuropathy including autoimmune diseases, chronic inflammatory demyelinating polyneuropathy (CIDP), vitamin B12 deficiency, alcoholism, and injury or pressure on the nerve. Finally, 580 T2DM patients were included in this study. According to the medical history, clinical manifestations, and nervous system examination (ankle reflex, acupuncture pain, vibration, pressure, temperature), there were 290 T2DM patients with DPN and 290 without DPN (Fig. 1).

### Clinical and laboratory examination

All patients completed 40 biochemical index tests by venous blood collected after fasting for 8 h. Metabolic index of thyroid gland including: thyroid stimulating hormone (TSH), free thyroxine (FT4), free triiodothyronine (FT3), four iodothyronine (T4), and triiodothyronine (T3) were measured by radioimmunoassay (North Institute of Biotechnology, Beijing). A set of lipid metabolism index contained total cholesterol (TC), triglycerides (TG), low-density lipoprotein (LDL), high density lipoprotein (HDL), apoprotein A (Apo A), apoprotein B (Apo B), apoprotein E (Apo E) and lipoprotein a (Lp-a). Myocardial enzyme covered aspartate aminotransferase (AST), lactate dehydrogenase (LDH), creatine kinase (CK), creatine phosphokinase-MB (CK-MB), alpha-hydroxybutyric dehydrogenase (α-HBDH), superoxide dismutase (SOD). Electrolyte related indicators contained anion gap (AG), calcium (Ca), kalium (K), phosphorus (P), chlorine (CL), magnesium (Mg), sodium (Na), CO2 combining power (CO2CP). Renal function index contained creatinine (Cr), blood urea nitrogen (BUN), uric acid (UA), glucose (GLU), retinol binding protein (RBP), glycated albumin (GA), Cystatin C (CysC). Biochemical test indexes related to liver function including prealbumin (PA), total protein (TP), albumin (ALB), alkaline phosphatase (ALP), globulin (GLB) and, alanine aminotransferase (ALT) were measured by clinical automatic biochemical analysis system (Hitachi LABOSPECT 008). At the same time, we collected the sex and age information of patients.

### Statistical analysis

The data of this study were analyzed with SPSS 22.0 statistical software. Continuous variables were expressed as median (interquartile range), while the categorical variables were expressed as the number of cases (n) and percentage (%). Mann–Whitney U test was used to compare the differences between two groups of continuous variables, and the Chi-square test was performed for the categorical variables. Spearman correlation analysis was used to analyze the relationship between other clinical indicators and FT4 in the DPN group. We stratified FT4 (< 14.03, 14.03–16.2, 16.2–18.3, ≥ 18.3 pmol/L), FT3 (< 4.25, 4.25–4.87, 4.87–5.51, ≥ 5.51 pmol/L), T4 (< 6.56, 6.56–7.65, 7.65–8.89, ≥ 8.89 μg/dL), T3 (< 1.02, 1.02–1.18, 1.18–1.37, ≥ 1.37 ng/mL) by quartile respectively and divided TSH (< 2.5, ≥ 2.5 μIU/mL) into two groups according to the other study^13^, to compare the prevalence of DPN between each group. Then the logistic regression analysis was used to study the relationship between the prevalence of DPN and TSH, FT4, FT3, T4, T3. *P* value < 0.05 was considered statistically significant.

### Ethics approval and consent to participate

This study was approved by the Ethics Committee of the First Affiliated Hospital of Xi'an Jiaotong University. All methods in the study were performed in accordance with relevant institutional/national/international guidelines. All individuals signed informed consent to participate in the study.

## Results

### Clinical and laboratory characteristics of the study participants

A total of 580 participants with T2DM were evaluated in our study, which consists of 378 (65.17%) male and 202 (34.83%) female, and the quartile of age was 53.5 (43–63) years. Among all participants, 290 were diagnosed with DPN, as DPN group; and the other 290 without DPN, as Non-DPN group. The characteristics of the patients were shown in Table 1. The sex ratio was the same in both groups. About the age, patients in Non-DPN group have a higher quartile than DPN group (54 (44–64) vs 52 (42–61) years, *P* = 0.004). A total of 12 indicators were statistically different between the two groups, among which AST, α-HBDH, Ca, SOD, Cr, UA, RBP, TP, ALB, ALT were higher in Non-DPN group than in DPN group (*P* < 0.05). For thyroid function, FT4 was lower in DPN group than in Non-DPN group (15.95 (13.9–17.8) vs 16.4 (14.3–18.83) pmol/L) (*P* < 0.05). There was no significant difference in TSH, FT3, T4 and T3.

**Table 1:** Clinical characteristics of T2DM patients with or without DPN.

Characteristics	Total (580)	Non-DPN (290)	DPN (290)	*P*
Gender (male)	378 (65.17%)	189 (65.17%)	189 (65.17%)	1.000
Age	53.5 (43–63)	54 (44–64)	52 (42–61)	0.004**
TSH	1.70 (1.12–2.58)	1.65 (1.06–2.50)	1.75 (1.19–2.67)	0.108
FT3	4.87 (4.25–5.51)	4.99 (4.33–5.56)	4.77 (4.19–5.42)	0.084
FT4	16.2 (14.03–18.3)	16.4 (14.3–18.83)	15.95 (13.9–17.8)	0.017*
T3	1.18 (1.02–1.37)	1.19 (1.05–1. 4)	1.17 (1.0–1.35)	0.087
T4	7.65 (6.56–8.89)	7.69 (6.57–8.99)	7.6 (6.54–8.80)	0.472
TG	1.51 (1.01–2.23)	1.49 (1–2.22)	1.53 (1.02–2.25)	0.850
TC	4.25 (3.51–4.9)	4.3 (3.49–4.91)	4.21 (3.52–4.84)	0.903
LDL	2.47 (1.91–3.09)	2.51 (1.89–3.11)	2.46 (1.93–3.08)	0.933
HDL	0.99 (0.85–1.21)	0.99 (0.84–1.22)	1.0 (0.88–1.2)	0.565
Apo A	1.19 (1.07–1.33)	1.20 (1.07–1.35)	1.18 (1.07–1.32)	0.400
Apo B	0.83 (.067–1.00)	0.85 (0.66–1.01)	0.83 (0.68–0.98)	0.924
Apo E	36.8 (29.18–48.15)	37.4 (29.8–49.7)	36.2 (28.2–46.8)	0.143
Lp (A)	117 (56.98–228)	123 (60–228)	105.7 (55–232)	0.506
AST	19 (15–25)	20 (16–26)	18 (15–23)	0.001**
LDH	182 (161–204)	182.5 (162–206)	179 (159–200)	0.170
CK	73 (53–100)	74.5 (55–103.5)	70 (50–94)	0.105
CK-MB	13 (10–16)	12 (10–16)	13 (10.65–16)	0.429
α-HBDH	146 (131–166)	148 (132.75–168)	142 (124.5–160.5)	0.015*
SOD	170.4 (154.1–188.4)	177.5 (161.38–194.63)	161.7 (146.4–177.9)	0.000***
AG	23.8 (21.2–26.6)	23.8 (21.5–26.6)	23.74 (20.91–26.65)	0.533
Ca	2.22 (2.15–2.29)	2.24 (2.16–2.31)	2.21 (2.14–2.27)	0.001**
K	3. 9 (3.69–4.18)	3.93 (3.73–4.19)	3.89 (3.65–4.15)	0.237
P	1.15 (1.02–1.28)	1.16 (1.03–1.28)	1.14 (1.01–1.26)	0.287
CL	103.4 (101–105.7)	103.45 (101.1–105.6)	103.3 (100.85–105.8)	0.842
Mg	0.9 (0.85–0.96)	0.9 (0.86–0.96)	0.9 (0.85–0.96)	0.985
Na	142 (140.3–144)	142 (141–144)	142 (140–144)	0.093
CO2CP	22 (19.9–24.3)	22 (19.8–24.3)	21.9 (19.95–24.2)	0.863
Cr	57 (47–67)	58 (48.75–67.25)	56 (46–66)	0.041*
BUN	5.26 (4.42–6.25)	5.25 (4.43–6.27)	5.29 (4.4–6.22)	0.803
UA	315 (257.45–374.78)	325.5 (267–386)	298.45 (249.28–366)	0.005**
GLU	6.93 (5.68–9.07)	6.8 (5.66–8.63)	7.18 (5.76–9.48)	0.113
RBP	52.4 (39.3–66.3)	54.35 (40.33–66.93)	47.2 (37.85–62.85)	0.049*
GA	19.2 (16.01–23.2)	18.95 (15.8–22.93)	19.75 (16.4–23.45)	0.135
CysC	0.82 (0.72–0.93)	0.82 (0.72–0.93)	0.82 (0.72–0.94)	0.789
PA	254.95 (215.6–291.95)	257.2 (221.13–292.88)	247.9 (205.43–291.53)	0.186
TP	66.8 (63.23–71.4)	67.5 (63.5–72.03)	66.2 (62.98–70.73)	0.015*
ALB	41.4 (39–44.2)	42.3 (39.1–45.03)	41 (38.6–43.23)	0.001**
ALP	71 (57–89)	70 (55.75–87)	72 (60–89.75)	0.107
GLB	25.3 (23.1–28)	25.45 (23.3–28.2)	25.15 (22.9–27.93)	0.290
ALT	20 (14.5–32)	22 (15–38)	19.7 (14.23–29.28)	0.020*

Normal reference range: TSH 0.25–5.0 μIU/mL, FT4 9.05–25.5 pmol/L, FT3 2.91–9.08 pmol/L, T4 4.2–13.5 μg/dL, T3 0.78–2.2 ng/mL. *P < 0.05; **P < 0.01; ***P < 0.001.

### Association between FT4 and clinical characteristics in DPN group

We used the Spearman correlation analysis to explore the relationship between other clinical indicators and FT4 in the DPN participants. As shown in the Table 2 FT4 was significantly negatively correlated with Cl, while the TP, PA, ALB, SOD, AG, Ca, UA, RBP, Apo A, Apo B, Apo E and TC were significantly positively associated with FT4 (*P* < 0.05). No significant association with FT4 were shown in Table S1.

**Table 2:** Association between FT4 and clinical characteristics among T2DM subject with DNP.

	TP	ALB	PA	SOD	AG	Ca	Cl
r value	0.187	0.233	0.159	0.164	0.217	0.240	− 0.143
*P*	0.002	0.000	0.024	0.007	0.000	0.000	0.016

	UA	RBP	Apo A	Apo B	Apo E	TC	
r value	0.187	0.285	0.144	0.148	0.160	0.128	
*P*	0.001	0.000	0.015	0.013	0.006	0.030	

### The prevalence of DPN among thyroid related indicators

**Figure 2:**
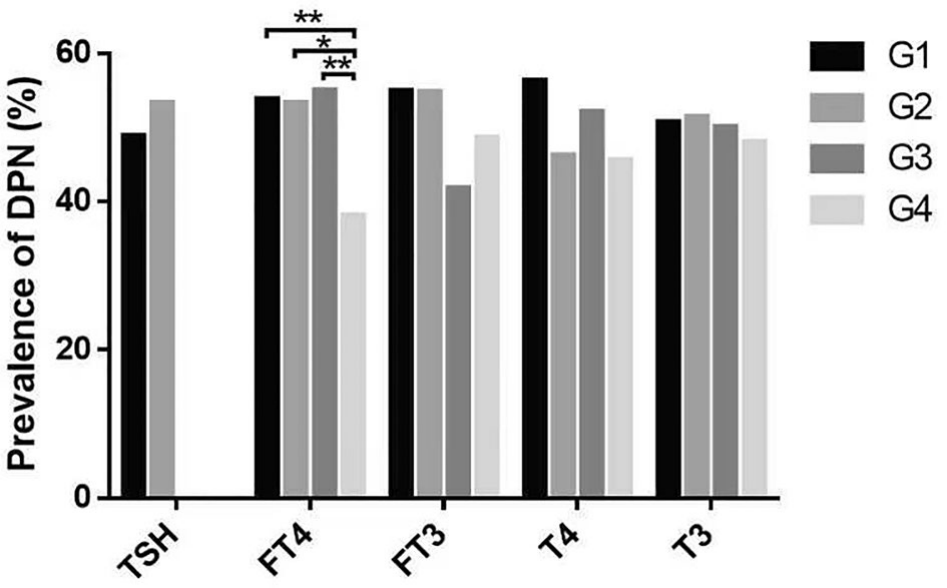
Prevalence of Diabetes Peripheral Neuropathy (DPN) in different levels of TSH groups, and prevalence of DPN among quartiles based on FT4, FT3, T4, T3 levels (*P < 0.05, **P < 0.01). TSH: Q1 < 2.5 μIU/mL, Q2 ≥ 2.5 μIU/mL; FT4: Q1 < 14.03 pmol/L, Q2 14.03–16.2 pmol/L, Q3 16.2–18.3 pmol/L, Q4 ≥ 18.3 pmol/L; FT3: Q1 < 4.25 pmol/L, Q2 4.25–4.87 pmol/L, Q3 4.87–5.51 pmol/L, Q4 ≥ 5.51 pmol/L; T4 Q1 < 6.56 μg/dL, Q2 6.56–7.65 μg/dL, Q3 7.65–8.89 μg/dL, Q4 ≥ 8.89 μg/dL; T3 Q1 < 1.02 ng/mL, Q2 1.02–1.18 ng/mL, Q3 1.18–1.37 ng/mL, Q4 ≥ 1.37 ng/mL (n = 580).

The total of 580 participants were divided into four groups based on the quartile of FT4 (Q1: < 14.03, Q2: 14.03–16.2, Q3: 16.2–18.3, Q4: ≥ 18.3 pmol/L), FT3 (Q1: < 4.25, Q2: 4.25–4.87, Q3: 4.87–5.51, Q4 ≥ 5.51 pmol/L), T4 (Q1 < 6.56, Q2: 6.56–7.65, Q3: 7.65–8.89, Q4: ≥ 8.89 μg/dL), T3 (Q1: < 1.02, Q2: 1.02–1.18, Q3: 1.18–1.37, Q4: ≥ 1.37 ng/mL), and two groups were dependent on the level of TSH (Q1: < 2.5, Q2: ≥ 2.5 μIU/mL), respectively. For each group, the last group was taken as the reference for comparative analysis. As shown in Fig. 2, the prevalence of DPN in FT4 Q4 group was significantly lower than that in the other three groups (Q1: 53.79%, Q2: 53.28%, Q3: 54.97%, Q4: 38.10%. *P* < 0.05). There was no significant difference in FT3, T4 and T3 groups.

### Association between thyroid functions and the prevalence of DPN

As shown in the Table 3, we further analyzed the relationship between the prevalence of DPN and TSH, FT4, FT3, T4, T3. Adjusted for gender and age, the results indicated that the crude ORs (95% CI) for DPN in FT4 Q1, FT4 Q2, and FT4 Q3 group were 0.529 (0.332 -0.843), 0.540 (0.336 -0.866), and 0.504 (0.318–0.800), respectively (*P* < 0.05) compared to those in FT4 Q4 group. Yet no significant association with DPN in TSH, FT3, T4 and T3 groups was found.

**Table 3:** Logistic regression analysis of thyroid hormone levels with DPN.

Characteristics	Groups	Number	B	OR (95% CI)	*P*
TSH	< 2.5	426	− 0.200	0.819 (0.563–1.191)	0.296
	≥ 2.5	154		Reference	
FT4	< 14.03	145	0.685	1.984 (1.237–3.181)	0.004**
	14.03–16.2	137	0.607	1.836(1.138–2.961)	0.013*
	16.2–18.3	151	0.739	2.093 (1.311–3.343)	0.002**
	≥ 18.3	147		Reference	
FT3	< 4.25	142	0.176	1.192 (0.744–1.910)	0.464
	4.25–4.87	146	0.158	1.171(0.734–1.870)	0.507
	4.87–5.51	146	− 0.321	0.725 (0.455–1.156)	0.177
	≥ 5.51	146		Reference	
T4	< 6.56	144	0.085	1.089 (0.683–1.736)	0.720
	6.56–7.65	146	0.095	1.100 (0.691–1.749)	0.688
	7.65–8.89	144	0.049	1.050 (0.660–1.672)	0.836
	≥ 8.89	146		Reference	
T3	< 1.02	144	0.338	1.402 (0.877–2.243)	0.158
	1.02–1.18	145	− 0.046	0.955 (0.599–1.523)	0.847
	1.18–1.37	144	0.2237	1.269 (0.798–2.018)	0.315
	≥ 1.37	147		Reference	

Adjusted for age, gender. *P < 0.05; **P < 0.01.

## Discussion

Thyroxine is a hormone synthesized and secreted by thyroid follicular epithelial cells, which is involved in regulating the metabolism of various substances in the body, including lipid synthesis and fat distribution. The study found that 25.7% of subclinical hypothyroidism (SCH) patients had metabolic syndrome (MS)^14,15^. In patients with T2DM with normal thyroid function, relatively high TSH levels were significantly associated with components of the metabolic syndrome. Patients with high TSH levels are prone to glucose metabolism disorders in the euthyroid people^16^. In addition, a number of studies have found that the level of thyroid hormone (TH) is related to a variety of chronic complications of diabetes, such as diabetic nephropathy and diabetic retinopathy^17,18^. It has also been reported that serum FT3 level is related to nerve conduction in diabetic patients, and low FT3 may be a potential risk factor of DPN^9^. It is known that low levels of FT4 are associated with metabolic syndrome and can aggravate insulin resistance, but whether low levels of FT4 are involved in the occurrence of DPN remains unclear. Therefore, we enrolled 580 inpatients with initial diagnosis of T2DM in this study. According to the medical history, clinical manifestations, and nervous system examination (ankle reflex, acupuncture pain, vibration, pressure, temperature), they were identified as DPN group and Non-DPN group. By analyzing the clinical characteristics, the relationship between thyroid function status determined by routine biochemical indexes of the two groups of patients and the risk factors of DPN were examined, which may provide new ideas for the prevention and treatment of clinical DPN.

The results demonstrated that the levels of AST, α-HBDH, SOD, serum Ca, Cr, UA, RBP, TP, ALB, ALT, and FT4 in DPN group were significantly lower than those in Non-DPN group, but they were within the reference range of normal population. Subsequently, FT4 was used as a variable for correlation analysis, and the results showed that F4 level was significantly positively correlated with Apo A, Apo B, Apo E, TC, TP, PA, ALB and Ca. Abnormal lipid metabolism is known to be one of the diagnostic criteria for metabolic syndrome, which is also a high-risk group for the occurrence of diabetes^19^. Many studies have found that abnormal lipid metabolism is a risk factor for the occurrence of DPN^20^. Lipid metabolism disorders can also affect vasodilation and contraction through vasoactive factors such as nitric oxide (NO) and endothelin-1(ET-1)^21^, thereby injure blood vessels or directly damage the structure and function of nerve cells, leading to or aggravating DPN. TC refers to the cholesterol contained in various lipoproteins in serum. Apo A is the main structural and functional protein of HDL, which regulates cholesterol reverse transport and inflammation by interacting with cell membrane receptors and activating lecithin cholesterol acyltransferase. TH affect lipid metabolism in many ways, such as lipid synthesis, mobilization, and degradation, so thyroid disease is often associated with significant dyslipidemia^22^. TH is not only associated with changes in the concentration of various lipid components, but also with changes in HDL function^23^. Therefore, when FT4 level is low in diabetic patients, the synthesis of serum HDL is blocked and its function is inhibited, which accelerates the formation of atherosclerosis and plaques. When the blood vessels of nutrient nerves are stenosis or even occluded, the occurrence of DPN was induced.

TP, ALB and PA have functions such as maintaining normal blood colloid osmotic pressure and pH value, transporting a variety of metabolite, regulating immunity and nutrition. It is known that TH level is affected by TBG, and we found that FT4 level is significantly positively correlated with TP, ALB and PA, which is considered to be related to the increase of microalbumin in diabetic patients. Ca ion was known to participate in the synthesis and release of neurotransmitters, maintain neuromuscular excitability and regulate the synthesis and secretion of hormones important ions. The lack of Ca in the human body could induce neuromuscular stress, and result in severe tetany. FT4 level is positively correlated with serum calcium level. When FT4 is at a low level, serum Ca level also decreases in parallel. Hypocalcemia affects nerve excitation conduction, which promotes the occurrence of DPN.

Finally, the relationship between thyroid function and DPN was analyzed by logistic regression. A stratified analysis of thyroid function in 580 euthyroid patients with T2DM found that the incidence of DPN was significantly increased when FT4 levels were lower than 18.3 pmol/L. We speculated that when FT4 was at a normal low level, it would have an impact on lipid metabolism, serum protein metabolism, oxidative stress and serum Ca level, and accelerate the occurrence of DPN. Patients with T2DM are more likely to develop SCH and hypoFT4 than healthy individuals^24,25^. It is very necessary to conduct thyroid function screening in T2DM patients. FT4 mainly regulates energy metabolism and is related to oxidative stress^26^. These results suggested that low-dose levothyroxine treatment can be given according to the thyroid function of patients in the early stage of T2DM, especially in clinical patients with low FT4. Through this treatment strategy, FT4 can be maintained at the upper limit of normal. Moreover, lipid metabolism disorder in T2DM would be improved and oxidative stress responses were inhibited, thus reducing the occurrence of DPN in T2DM.

However, this study has its limitations. First of all, gender representation was not a random division of euthyroid diabetics into subgroups. Secondly, although the patients enrolled in the current study were new-diagnosed diabetes, the patient's medical history could not be precisely determined as the patient was from a different region.

Collectively, the level of FT4 was negatively correlated with the prevalence of DPN in euthyroid T2DM patients. It is expected to provide a new strategy for the diagnosis and treatment of DPN.

### Supplementary Information

Supplementary Table S1.

## Data Availability

All data generated or analyzed during this study are included in this article. Further inquiries can be directed to the corresponding author.
