# Supplementary material for: Association between thyroid function and diabetes peripheral neuropathy in euthyroid type 2 diabetes mellitus patients
Source: Sci Rep. 2023 Aug 18;13:13499. doi: 10.1038/s41598-023-40752-y (PMC10439138; doi:10.1038/s41598-023-40752-y)
Supplement: Supplementary file 1 — Supplementary Table S1. [file 41598_2023_40752_MOESM1_ESM.docx]

Table. S 1. Association between FT4 and clinical characteristics among T2DM subject with DNP.

|  | | Gender | Age | | GLB | ALT | | AST | | ALP | LDH | CK |
| --- | --- | --- | --- | --- | --- | --- | --- | --- | --- | --- | --- | --- |
| r value | | 0.018 | -0.030 | | 0.048 | 0.003 | | 0.023 | | -0.026 | 0.080 | 0.105 |
| *P* | | 0.762 | 0.613 | | 0.423 | 0.954 | | 0.699 | | 0.658 | 0.339 | 0.211 |
|  | |  |  | |  |  | |  | |  |  |  |
|  | | CK-MB | | α-HBDH | LDL | CO2CP | | TG | | HDL | Cr | K |
| r value | | -0.030 | | 0.113 | 0.089 | -0.071 | | 0.108 | | 0.023 | 0.000 | 0.068 |
| *P* | | 0.720 | | 0.174 | 0.137 | 0.236 | | 0.069 | | 0.694 | 0.997 | 0.257 |
|  |  | |  | |  | |  |  |  |  |  |  |
|  | | P | Mg | | Na | BUN | | GLU | | GA | Lp(A) | Cys C |
| r value | | 0.031 | 0.066 | | 0.059 | 0.076 | | 0.036 | | -0.124 | 0.010 | -0.028 |
| *P* | | 0.682 | 0.268 | | 0.327 | 0.198 | | 0.559 | | 0.084 | 0.864 | 0.632 |
